# Long-Lasting LTP Requires Neither Repeated Trains for Its Induction Nor Protein Synthesis for Its Development

**DOI:** 10.1371/journal.pone.0040823

**Published:** 2012-07-11

**Authors:** Agnès Villers, Emile Godaux, Laurence Ris

**Affiliations:** Department of Neurosciences, University of Mons, Mons, Belgium; University of Toronto, Canada

## Abstract

Current thinking about LTP triggered in the area CA1 of hippocampal slices is ruled by two “dogmas”: (1) A single train of high-frequency stimulation is sufficient to trigger short-lasting LTP (1 – 3 h), whereas multiple trains are required to induce long-lasting LTP (L-LTP, more than 4 h). (2) The development of the late phase of L-LTP requires the synthesis of new proteins. In this study, we found that a single high-frequency train could trigger an LTP lasting more than 8 h that was not affected by either anisomycin or cycloheximide (two inhibitors of protein synthesis). We ascertained that the induction of this L-LTP made use of the same mechanisms as those usually reported to be involved in LTP induction: it was dependent on NMDA receptors and on the activation of two “core” kinases, CaMKII and PI3K. These findings call into question the two “dogmas” about LTP.

## Introduction

Observations on patient H.M. have led to the undisputed idea that two different types of memory exist: short-term memory (minutes to a few hours) and long-term memory (days, weeks, years) [1]. From a comprehensive review of experiments carried out on animals, Davis and Squire [2] concluded that short-term memory was independent of protein synthesis whereas long-term memory was prevented by protein-synthesis inhibitors (such as anisomycin) when administered before or just after training. Although very popular, this conclusion has recently been questioned [3]–[6].

At the cellular level, memories are nowadays believed to be encoded in neuronal networks in the brain by synaptic plasticity – more specifically, by changes in the strength of the synapses. One of the most studied types of synaptic plasticity is long-term potentiation (LTP) elicited in the CA1 region of hippocampal slices through stimulation of the Schaffer collaterals. Current thinking about LTP is ruled by two widely accepted “dogmas”. (1) In analogy with memory, LTP is believed to consist of two different temporal phases, each requiring a different type of triggering stimulation. A relatively short-lasting LTP (1–3 h) (S-LTP) is induced with a single train of high-frequency stimulation (100 Hz, 1 s), whereas triggering a long-lasting LTP (L-LTP) (more than 4 h) requires repeated trains of stimulation (3 or 4, 5 or 10 min apart) [7]. (2) Also in analogy with memory, the late phase of L-LTP is believed to depend on a protein synthesis process triggered by the LTP-inducing stimulus whereas the early phase of L-LTP and the S-LTP induced by a single train would rely on post-translational modifications of pre-existing proteins and on incorporation of spare AMPA receptors into the postsynaptic density of the dendritic spines [8].

Here, we found that an L-LTP lasting more than 8 h could be induced with a single train (in disagreement with dogma 1), in presence of inhibitors of protein synthesis (in disagreement with dogma 2). Analysis of the properties of this L-LTP showed that it was induced through the classical pathways usually involved in the induction of an LTP triggered in the CA1 region of the hippocampus. It was dependent on NMDA receptors, alpha-calcium-calmodulin-kinase II (α-CaMKII) and PI3-Kinase (PI3-K) for its induction. The independence of the late phase of this L-LTP from new protein synthesis was confirmed by several experiments and is discussed in relation to recent literature.

## Results

### A single train of high frequency stimulation can induce a very-long-lasting LTP

**Figure 1:**
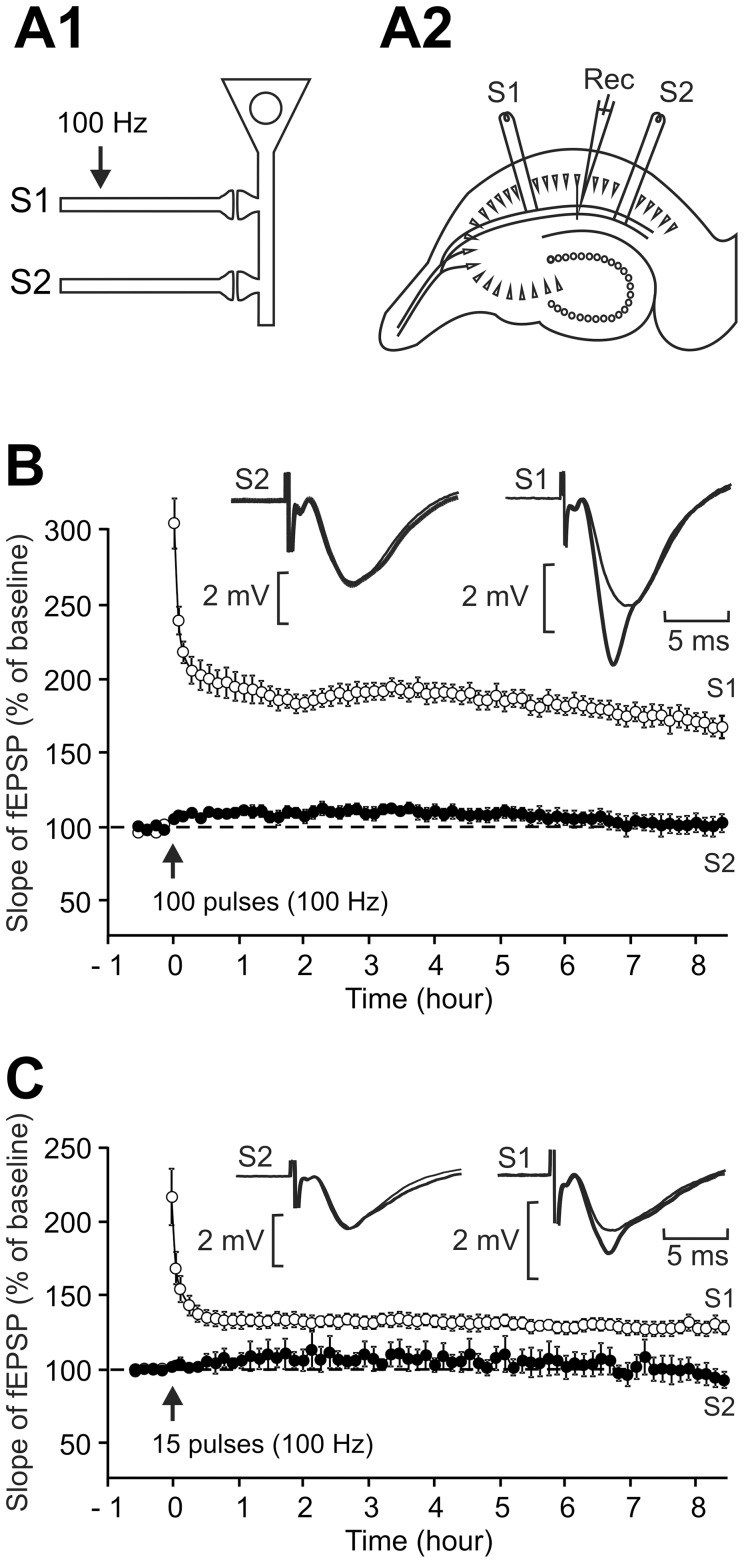
Induction of an L-LTP lasting for more than 8 h using a single train of high-frequency stimulation. (A1, A2) Sketch showing the two independent synaptic inputs S1 and S2 to the same neuronal population. In each slice, two stimulating electrodes (S1 and S2) were put in place. S1 pathway was used to induce LTP, while S2 pathway acted as a control. (B, C) LTP induced by a single train of high-frequency stimulation (100 Hz) consisting of either 100 pulses (B, n = 6) or 15 pulses (C, n = 6). Each graph shows the time course of the fEPSP slope from 30 min before to 8 h30 after LTP induction. Sample fEPSP traces from individual experiments are shown in the insert for the S1 and the S2 pathways. They were recorded just before the LTP induction (thin traces) and at the end of the experiment (thick traces).

Repeated stimulation has been mechanistically taken as a requirement for late-LTP to occur. However, studies carried out in rat hippocampal slices have revealed that a single tetanus could also trigger a long-lasting LTP [9]–[12]. Further, in C57BL/6 mice prolonged theta stimulation (30 s, 5 Hz) [13] and a single brief theta-burst stimulation (3 s) [14] induce a long-lasting LTP. In the present experiments, we found that a single train of high frequency stimulation (100 Hz, 1 s) could induce a very long-lasting LTP in mouse hippocampal slices maintained in interface (Fig. 1B). In the very stable recording conditions created by using the Edinburgh temperature controller system and bipolar cluster electrodes (FTC), a train of 100 impulses at 100 Hz applied through S1 triggered a long-lasting LTP in the synapses tested via S1. Eight and a half hours after induction, the slope of the fEPSP was 168.4±7.3% of the baseline level (n = 6), while the strength of the synapses tested via S2 remained unchanged (102.5±6.6% of baseline, n = 6, P<0.001).

### Reducing the number of pulses in the train reduces the amplitude but not the duration of LTP

There is a common and widely accepted hypothesis that LTP, like memory, has distinct stages or phases but one can wonder whether the different time courses of LTP would rather only reflect quantitative differences depending on the tetanization strength.

In recent papers, the weakest stimulation enabling the induction of a short-lasting LTP was reduced to 21 pulses [15], [16] whereas Bortolotto et al [17] showed that a further smaller number of pulses (8) was sufficient to induce metaplasticity. So, we also tried to reduce the number of pulses in the train of stimulation. We found that even a short train of 15 impulses induced a long-lasting LTP (Fig. 1C). The fEPSP slope was 127.9±4.5% of baseline 8 h 30 min after induction in synapses tested via S1 (n = 6) while it remained unchanged in synapses assessed via S2 (92.4±5.3%, n = 6, P<0.001). However, the amplitude of the LTP induced with 15 pulses was smaller than that obtained with a 1-sec train (P<0.005). The LTP induced by a short (15 pulses) or a longer (100 pulses) high-frequency train did not differ from each other in their duration but in their amplitude (compare Fig. 1B and Fig. 1C).

### Increased ease for inducing a long-lasting LTP is not dependent on previous NMDA activation

**Figure 2:**
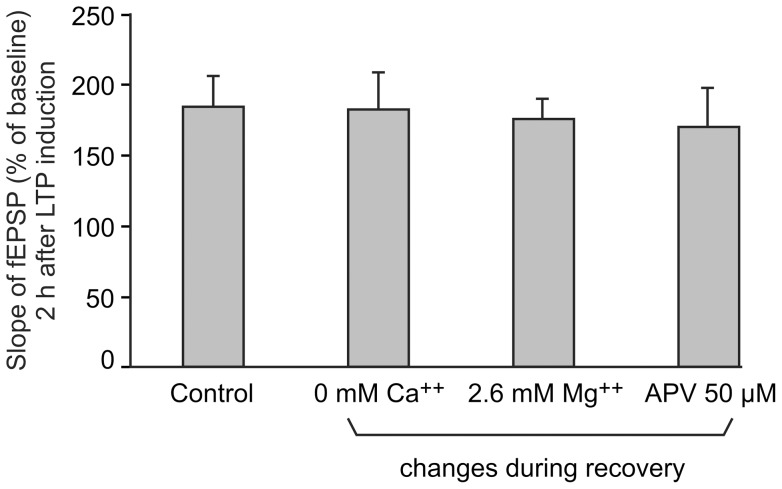
Previous NMDA activation is not required for the increased ease in inducing a long-lasting LTP. The bars represent the fEPSP slopes obtained 2 h after LTP induction by a single train (100 Hz, 1 s) when dissection and recovery occurred during perfusion of varied ACSF solutions whereas normal ACSF was applied during LTP induction and LTP maintenance. Dissection and recovery of slices were carried out in ACSF either of normal composition (n = 3), without Ca^++^ (n = 2), with 2.6 mM Mg^++^ (n = 2) or added with 50 µM APV, an NMDA receptor inhibitor (n = 3).

We wondered whether the capacity to induce a long-lasting LTP with a single train was not due to previous activation of the NMDA receptors during dissection and recovery. To test this hypothesis, NMDA receptor activation was prevented during dissection and recovery by perfusing the slices during these two periods with modified ACSF (either without Ca^++^, or with increased [Mg^++^], or added with APV) whereas usual ACSF was used during LTP induction and LTP maintenance. As shown in figure 2, neither ACSF without Ca^++^, nor ACSF with 2.6 mM Mg^++^ nor ACSF added with APV impaired the LTP induced by 1 train (One-way ANOVA, F(3,6)  = 0.0736, P = 0.972).

### Anisomycin and cycloheximide are two potent protein-synthesis inhibitors when applied on slices

**Figure 3:**
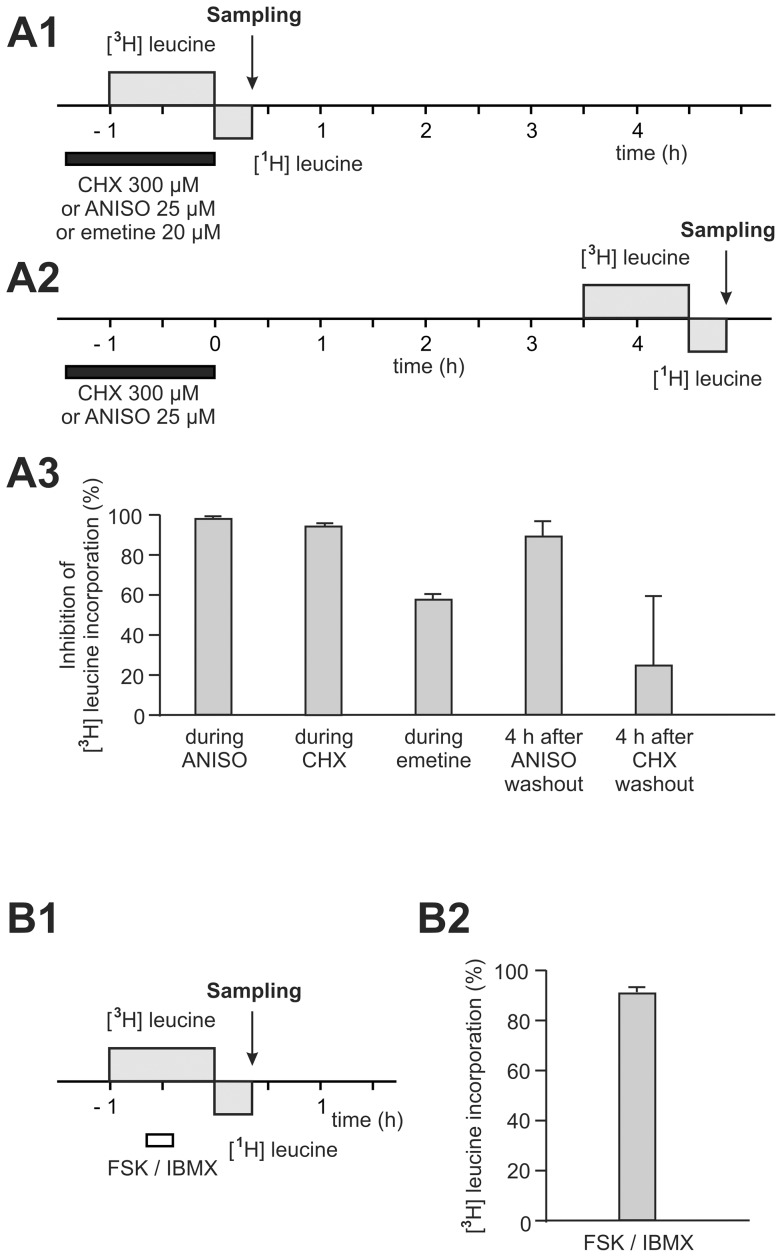
Cycloheximide and anisomycin inhibit protein synthesis in hippocampal slices. (A1–A2) Mice hippocampal slices were incubated in an interface chamber and CHX (300 µM), anisomycin (25 µM) or emetine (20 µM) was added for 85 min. [^3^H]-leucine (1 µCi/ml) was introduced for 1 hour during (A1) or 4 h after (A2) protein synthesis inhibitor application. We calculated the percentage of inhibition at each time point by determining the ratio between the TCA-precipitable radioactivity of each treated sample (set of 7 individual slices) and that of the corresponding control sample. (A3) Percentage of inhibition of protein synthesis during anisomycin (ANISO), cycloheximide (CHX) and emetine application and 4 h after ANISO or CHX application. (B1–2) Percentage of [^3^H]-leucine incorporation in slices submitted to forskolin and IBMX during 15 minutes. This percentage was calculated by determining the ratio between TCA-precipitable radioactivity of treated sample and that of the corresponding control sample.

Before testing whether L-LTP was protein synthesis-dependent, we assessed the effectiveness of known protein-synthesis inhibitors when applied in our experimental conditions. The effectiveness of anisomycin, cycloheximide and emetine in inhibiting protein synthesis was checked in slices that were maintained artificially alive in the same conditions as those used to evoke L-LTP. The slices were perfused in an interface chamber and the drug (either anisomycin or cycloheximide or emetine) was added to the perfusion medium for 85 min. [^3^H] leucine (1 µCi/mL) was introduced 25 min after the start of the 1 h 25-drug application. The radioactive nucleotide was washed out by perfusion for 20 min while the non-radioactive isotope of leucine was present (Fig. 3A1). We calculated the percentage of inhibition by determining the ratio between the TCA-precipitable radioactivity of the drug-treated sample (set of 7 individual slices) and that of the corresponding control sample. Anisomycin (25 µM) and cycloheximide (300 μM) inhibited [^3^H] leucine incorporation into the proteins of the slices by 97.3±1.0% (n = 3) and by 96.5±1.4% (n = 3), respectively (Fig. 3A3). Emetine when applied at the concentration usually used in slices (20 µM) was not found to be a powerful protein-synthesis inhibitor. In the presence of emetine, [^3^H]-leucine incorporation into the proteins of the slice was only inhibited by 65±1% (n = 2) (Fig. 3A3).

**Figure 4:**
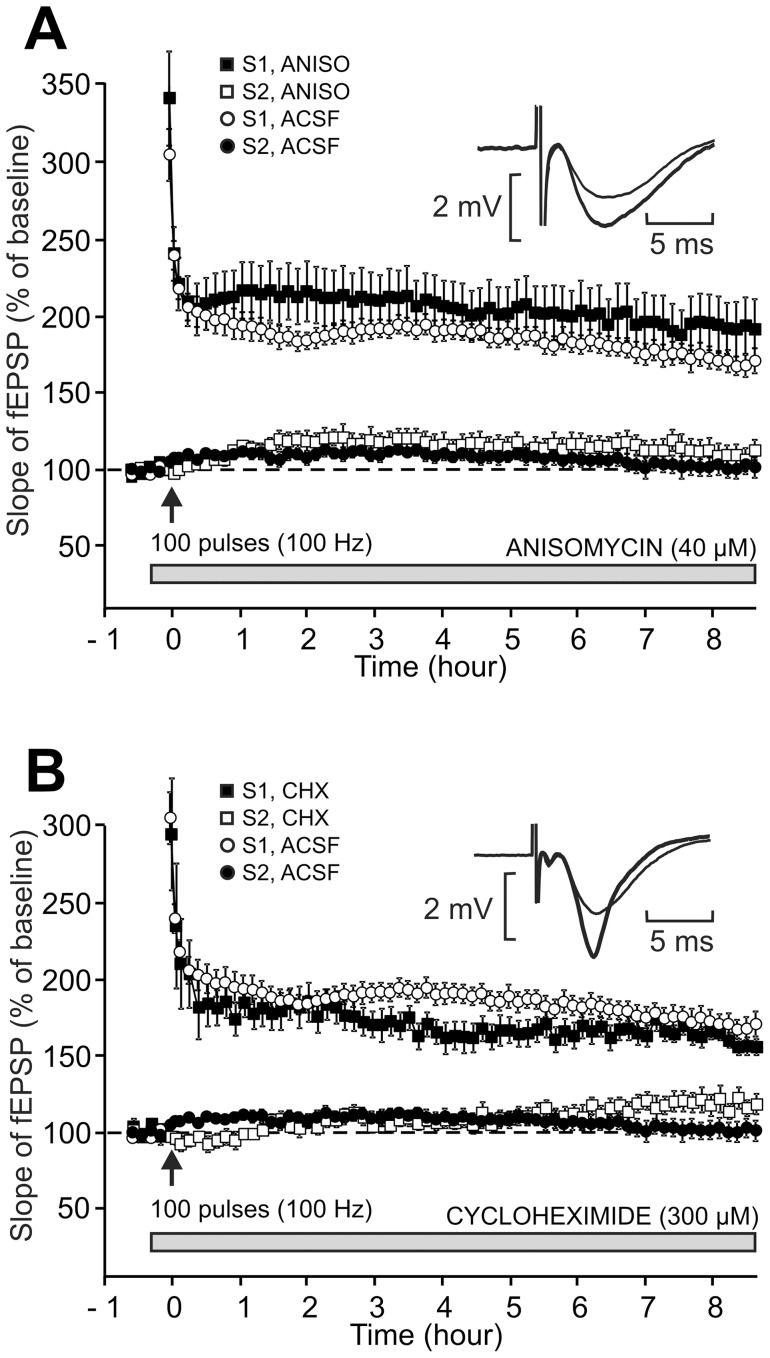
Lack of effect of anisomycin and cycloheximide, two translational inhibitors of protein synthesis, on L-LTP. In the two parts of the figure (A, B), either drug was applied starting 20 min before the induction of LTP on the S1 pathway till the end of the experiment. LTP was induced using a single high-frequency stimulation train (100 Hz, 1 s). In each part of the figure, the time courses of the fEPSP slope in the S1 pathway and S2 pathway (control), both in the presence and in the absence of the protein-synthesis inhibitor used, are displayed. Sample fEPSP traces from individual experiments are shown in the insert; they were recorded in the presence of the drug, before (thin traces) and 8 h30 after (thick traces) LTP induction. (A) Lack of effect of anisomycin at a dosage of 40 µM. (B) Lack of effect of cycloheximide at a dosage of 300 µM.

We tested the reversibility of anisomycin and cycloheximide (CHX) by measuring [^3^H] leucine incorporation 4 h after drug washout (Fig. 3A2). Four hours after washout of CHX, [^3^H] leucine incorporation was only inhibited by 24.5±34.8%, indicating the reversibility of CHX action. That is why we have added the drug during the whole experiment (Fig. 4B). On the other hand, 4 h after anisomycin washout, the protein synthesis was still reduced by 89.0±7.2%.

Anisomycin is thus a powerful inhibitor of the basal protein synthesis. However, it could not be ruled out that the protein synthesis triggered by the inductive stimulus of L-LTP would be so large that anisomycin (25 µM) would not be able to suppress it. We put this hypothesis to the test, using a chemical induction of LTP in order to affect a great number of synapses. LTP was induced by a 15-min application of forskolin (50 µM) and IBMX (30 µM) (Fig. 3B1). Thirty minutes later, the incorporation of [^3^H] leucine was not increased (90.0±0.7% of control protein synthesis, Fig. 3B2), showing that the induced protein synthesis was surely not quantitatively significant.

### The long-lasting LTP induced by a single train is not dependent on de novo protein synthesis

Sajikumar et al. [12] showed that a late-LTP could be induced by a single train in rat CA1 hippocampal neurons. They also demonstrated that this late-LTP was dependent on de novo protein synthesis as it was blocked by anisomycin and emetine. Fonseca et al. [18] also showed a long-lasting LTP induced by a single train and related the dependence on de novo protein synthesis to the frequency of the test stimulation. The more rapid the frequency of the test stimulation, the more the LTP was sensitive to protein-synthesis inhibitors.

We checked whether the LTP induced by a single train was sensitive to the two protein-synthesis inhibitors most used in slices, anisomycin (40 µM) and cycloheximide (300 µM). The delivery of either drug started 20 min before the train of stimulation and was maintained during the entire experiment. The fEPSP slopes measured at the end of the experiments were not different – whether anisomycin was applied or not (187.5±17.4%, n = 6, in the presence versus 168.4±7.3%, n = 6, in the absence of the drug, P = 0.335) (Fig. 4A). Similarly, the fEPSP slopes measured at the end of the experiments were not different in the presence or in the absence of cycloheximide (154.9±3.8%, n = 6 versus 168.4±7.3%, n = 6, P = 0.129) (Fig. 4B).

### Extension of the recovery period does not change the sensitivity of the LTP to anisomycin

In a methodology paper, Sajikumar et al [11] recommended to wait at least 4 h after dissection before inducing LTP. According to these authors, this long resting period provides a more stable recording but also allows the slices to reach a metabolic stability and might lead to a reduction of the level of the plasticity-related proteins (PRPs) close to zero. In a recent paper, Redondo et al [15] also used this long resting period before inducing a protein synthesis-dependent LTP.

**Figure 5:**
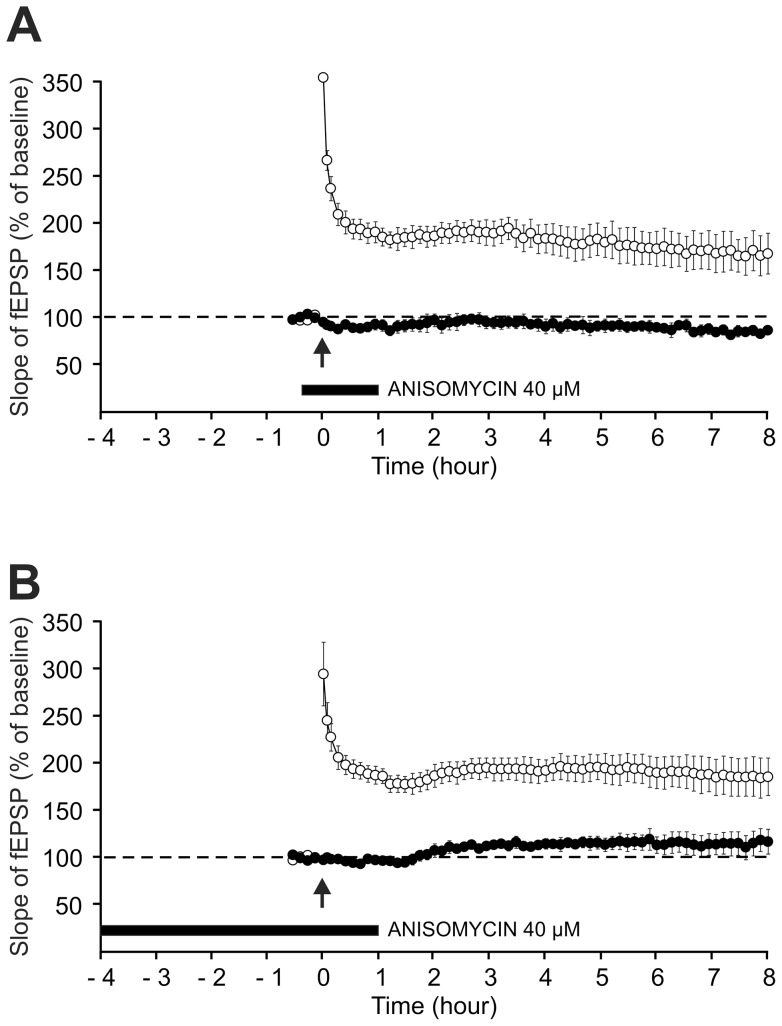
Plasticity-related proteins are synthesized neither during LTP induction nor during the recovery period. For these experiments, the recovery period was extended to 4 h. LTP was triggered using a single high-frequency stimulation train (100 Hz, 1 s). Open circles correspond to the fEPSP slopes measured on the potentiated pathway, whereas filled circles represent the fEPSP slopes measured on the control pathway. (A) Anisomycin (40 µM) was applied starting 20 min before till 1 h after LTP induction (n = 4). (B) Slices were incubated in anisomycin (40 µM) during their recovery period till 1 h after LTP induction (n = 4).

We therefore applied anisomycin after a recovery period in interface of 4 h. In order to diminish the risk of side effects of weakly reversible drug, anisomycin was further applied only for 1 h after the induction of LTP. As shown in figure 5A, the potentiation reached 8 h after the train in such conditions was not different from that obtained when the recovery period lasted 1.5 h. Eight hours after the train, the potentiation was still at 168.2±21.6%, a value not statistically different from that obtained with a 1.5 h recovery period (194.3±18.4%, t-test, P = 0.4).

### Plasticity-related proteins were not synthesized during the recovery period

It has been repeatedly claimed that the development of the late phase of L-LTP necessitates the synthesis of plasticity-related proteins (PRPs). We demonstrated above that, in our experimental conditions, these PRPs were not synthesized during the LTP induction or after it. A possibility remained that these PRPs would be synthesized during the recovery period. To assess the hypothesis, we pre-incubated slices during 4 h with anisomycin before inducing LTP (Fig. 5B). Here again, the level of potentiation reached 8 h after induction (186.1±19.9%) was not different from that observed in control experiments (194.3±18.4% after 1.5 h recovery period and 168.2±21.6% after 4 h recovery period (one-way ANOVA, F(2,11)  = 0.44, P = 0.65)).

### The long-lasting LTP induced by a single train is dependent on NMDA receptors, α-CaMKII autophosphorylation and PI3-K

In our experimental conditions, the L-LTP triggered by a single train was found to be surprisingly protein synthesis-independent. We next ascertained that it shared the main properties of the L-LTP reported in the literature, i.e. its dependence (1) on NMDA receptors, and (2) on two “core” kinases: α-CaMKII and PI3-K [19].

**Figure 6:**
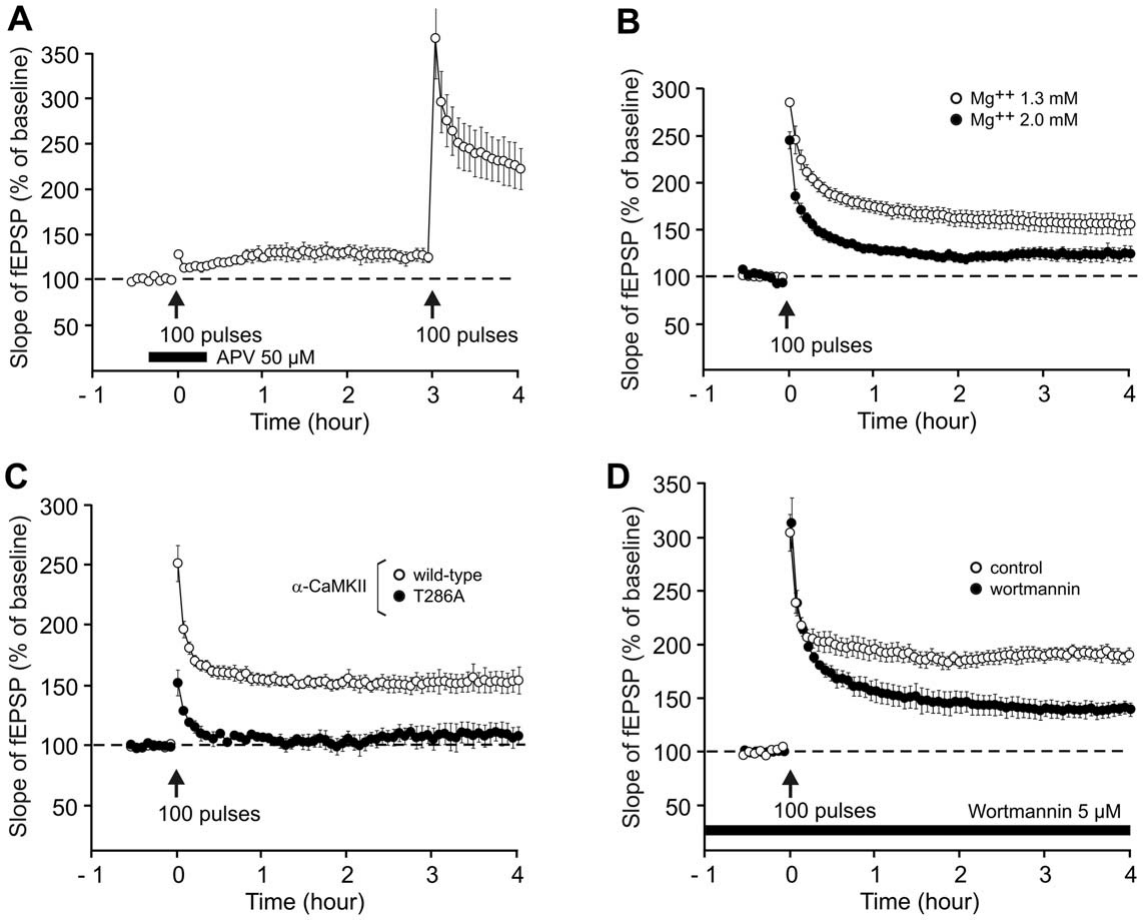
NMDA receptors, α-CaMKII autophosphorylation and PI3K are required to induce a long-lasting LTP by a single train. Each graph shows the time course of the fEPSP slope after LTP induction by a single train of high-frequency stimulation (100 Hz, 1 s). (A) APV (50 µM) was applied starting 20 min before till 20 min after LTP induction. Following washout of the drug, LTP was successfully triggered by a second inducing stimulus, 3 h after the first one (n = 3). (B) Dissection, recovery and recordings were carried out in ACSF containing either 1.3 mM Mg^++^ and 2.5 mM Ca^++^ (open circles, n = 16) or 2 mM Mg^++^ and 2.2 mM Ca^++^ (filled circles, n = 4). Other ACSF components were unchanged. (C) LTP was induced in α-CaMKII T286A mutants (filled circles, n = 3) or wild-type mice (open circles, n = 6). (D) Slices were treated with wortmannin (5 µM), a PI3K inhibitor, starting 1 h before LTP induction till the end of the experiment (filled circles, n = 4) or incubated in normal ACSF (open circles, n = 6).

When APV (50 µM), an NMDA receptor competitive antagonist, was added at the moment of induction, it caused a severe reduction of LTP. One hour after the train, we observed a residual potentiation of 124.1±4.4% not statistically different from the baseline (P = 0.09) (Fig. 6A). As shown in figure 6A, after washing the drug, LTP could be induced normally in the same slices.

To further investigate the NMDA-dependence of LTP, we increased the Mg^++^ concentration. We modified the ACSF composition to alter the ratio between calcium and magnesium concentrations (from 2.5 mM Ca^++^/1.3 mM Mg^++^ to 2.2 mM Ca^++^/2 mM Mg^++^). Two hours after its induction, the LTP was reduced to 119.7±4.9% in the presence of 2.2 mM Ca^++^/2 mM Mg^++^, compared to 162.5±8.5% when the Ca^++^ and Mg^++^ concentration were 2.5 mM and 1.3 mM, respectively (t-test, P = 0.025, Fig. 6B).

Using α-CaMKII autophosphorylation-deficient (T286A) mutant mice, kindly provided by Dr. Karl Peter Giese (King's College London, Institute of Psychiatry), we also demonstrated that LTP was dependent on α-CaMKII (Fig. 6C). α-CaMKII-dependent LTP is generally accepted to involve enhanced synaptic trafficking of GluR1 subunits, as well as phosphorylation of GluR1 at Ser831 that enhances AMPA receptor conductance [20], [21]. In T286A mice, the slope of the fEPSP measured one hour after the train was 107.3±3.2% of the baseline level, a value not different form that observed in the control synapses (t test, P = 0.293, Fig. 6C).

Next, we tested whether LTP induced by 1 train was dependent on PI3-K, a kinase required for AMPA receptor insertion during LTP [22]. Application of wortmaninn (5 µM), an inhibitor of PI3-K, was started one hour before LTP induction and continued till the end of the experiment. Four hours after the train, the potentiation was reduced to 139.6±6.2% instead of 190.6±6.7% without the drug (Fig. 6D, P<0.001).

### Increasing the basal frequency of stimulation causes a decrease in the potentiation induced by a single train

In rat hippocampal slices, Fonseca et al [18] found that the sensitivity of L-LTP to protein-synthesis inhibitors was dependent on the protocol used for the test-pulse stimulation. In their study, they showed that when the test-pulse frequency was 0.1 Hz, the early phase of LTP, like its late phase, became sensitive to anisomycin applied around the time of induction. Fonseca et al [18] also demonstrated that at that test frequency (0.1 Hz), anisomycin applied 1 h after induction also caused a decay in the late phase of L-LTP, provided the test pulses were stopped after induction and resumed during application of the protein-synthesis inhibitor. Their finding is consistent with the idea that synaptic activity increases the turnover of proteins necessary for LTP, thereby modulating LTP stability. In another study, Volianskis and Jensen [23] showed that what they called “transient LTP” could be stored for more than 6h but decayed as a result of synaptic activation. The decay time of that t-LTP was inversely related to the frequency of stimulation during the post-tetanic test period.

**Figure 7:**
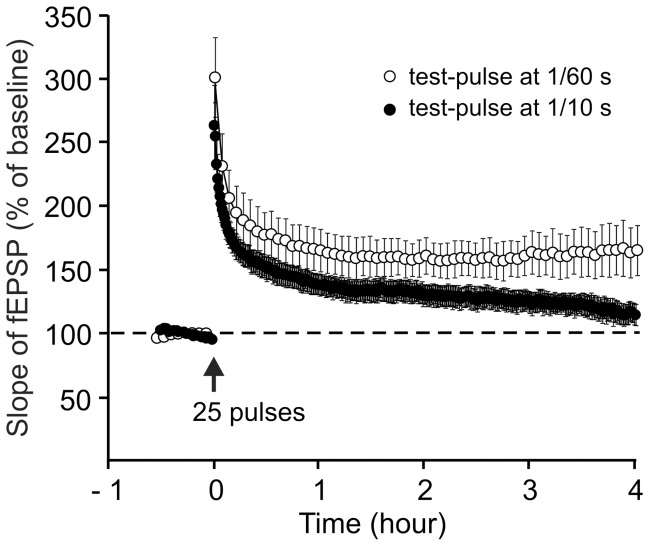
LTP induced by a single train decreases when test stimulation frequency is increased. Comparison of the time courses of the fEPSP slope after LTP induction by a single train of high-frequency stimulation (100 Hz, 0.25 s) when the test stimulation was delivered every 60 s (open circles, n = 5) or every 10 s (filled circles, n = 6). For clarity, only the data related to the tetanized pathways are shown.

In this study, LTP, tested in mouse hippocampal slices, was found not to be dependent on protein synthesis. It was thus interesting to investigate whether increasing the test-pulse frequency could modify the maintenance phase of LTP. We found that if the Schaffer collaterals were stimulated every 10 sec, the potentiated response decreased progressively. Four hours after the train, the slope of the fEPSP was not different from baseline (Fig. 7, 115.4±10.4%, paired t-test, P = 0.09).

### The proportion of homomeric GluR1 AMPA receptors did not change after LTP induction

Three types of AMPA receptors are present in the synaptic membrane: homomeric GluR1 receptors (GluR1), heteromeric GluR1-GluR2 receptors (GluR1-GluR2) and heteromeric GluR2-GluR3 receptors (GluR2-GluR3). In cultured cells, it has been demonstrated that these receptors are incorporated into the membrane in an activity-dependent manner for GluR1 and GluR1-GluR2 and thanks to a constitutive process for GluR2-GluR3 (For a review [24]). To investigate whether the relative proportions of the three types of AMPA receptors are modified during LTP, we used NASPM, a drug which selectively inhibits GluR2-lacking AMPA receptors, i.e. homomeric GluR1 receptors (GluR1).

**Figure 8:**
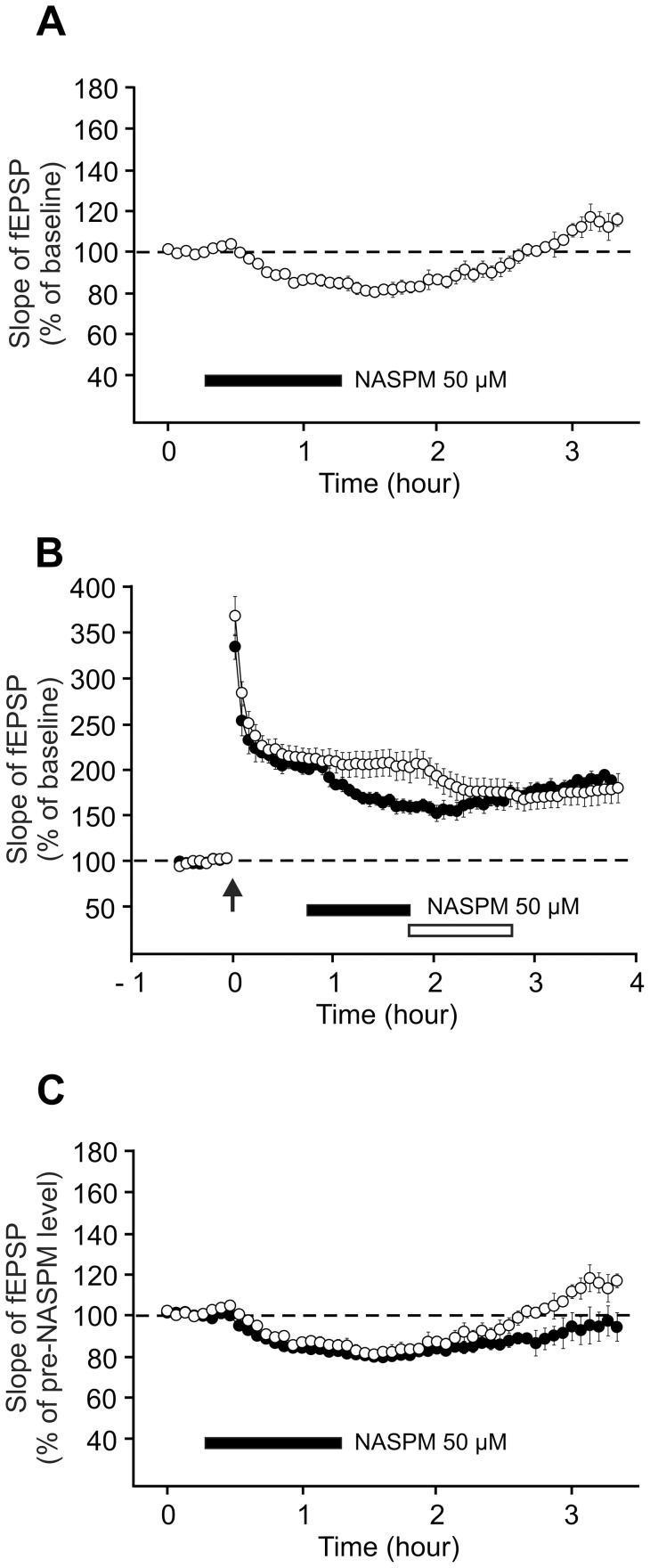
The proportion of homomeric GluR1 AMPA receptors in the post-synaptic sites is not altered following LTP induction. In these experiments, slices were treated with a 1-hour perfusion of NASPM (50 µM), an inhibitor of GluR2-lacking AMPA receptors. (A) NASPM was applied on a control pathway (n = 7). Baseline was calculated on the 20 minutes prior to the drug perfusion. (B) NASPM was added either 45 minutes (filled circles, n = 3) or 1 h45 (open circles, n = 4) after LTP induction by a single train of high-frequency stimulation (100 Hz, 1 s). Only data related to the tetanized pathways are shown. (C) Comparison of the decrease in the fEPSP slope induced by a 1-hour application of NASPM when the drug is applied on control synapses (open circles, n = 7) or on potentiated synapses (filled circles, n = 7). To facilitate the comparison, the fEPSP slope is expressed in percentage of the mean fEPSP amplitude measured (over a period of 20 min) before the application of the drug. In view of their similarity, the data related to the two time windows of application of NASPM during LTP maintenance (see B) were pooled.

Application of NASPM (50 µM) induced a 20% reduction of the fEPSP slope in basal synaptic transmission (Fig. 8A and Fig. 8C). When applied after LTP induction (Fig. 8B), NASPM induced a similar reduction in the response: the potentiation was decreased from 202.6±8.2% to 158.1±7.3% (20% reduction, Fig. 8C). This effect was reversible: about 30 minutes after the washout of the drug the response came back to its initial level. When applied 2 h after induction, the drug reduced the response again by around 20% (from 206.0±14.6% to 167.6±12.6%).

These results show that, during LTP, there is no change in the ratio between the number of homomeric GluR1 receptors and the number of heteromeric GluR2-containing receptors (GluR1-GluR2 + GluR2-GluR3).

### LTP induced by multiple trains is not dependent on de novo protein synthesis either

We have shown that the L-LTP induced by a single HFS train (100 Hz, 1 sec) did not require protein synthesis. However, this does not rule out the possibility that an L-LTP induced by repeated trains of stimulation would be protein-synthesis dependent. We thus checked the effect of a protein-synthesis inhibitor on the L-LTP induced by 4 trains (100 Hz, 5 min apart). Anisomycin (40 µM) was applied 20 min before the four trains of stimulation and was maintained during the entire experiment.

**Figure 9:**
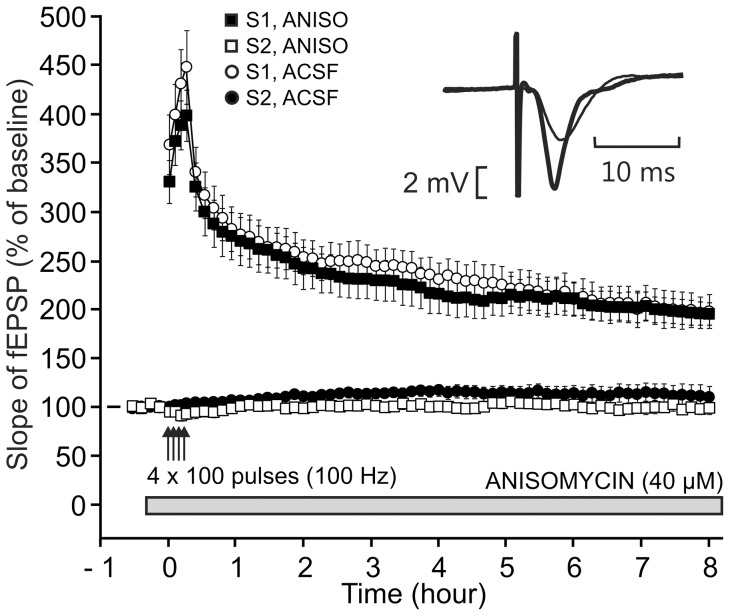
Lack of effect of anisomycin on L-LTP induced by four trains of high-frequency stimulation. Anisomycin (40 µM) was applied starting 20 min before the induction of LTP on the S1 pathway till the end of the experiment. LTP was induced using four high-frequency stimulation trains (100 Hz, 1 s, 5 min apart). The time courses of the fEPSP slope in the S1 pathway and S2 pathway (control), both in the presence and in the absence of the drug, are displayed. Sample fEPSP traces from an individual experiment are shown in the insert; they were recorded in the presence of the drug, before (thin traces) and 8 h after (thick traces) LTP induction.

As shown in Figure 9, the fEPSP slopes measured at the end of the experiments were not different – whether anisomycin was applied or not (195.5±11.5%, n = 7, in the presence versus 198.1±17.6%, n = 8, in the absence of the drug, P = 0.907). The strength of the synapses tested via S2 remained unchanged in the presence of anisomycin (99.7±6.6% of baseline).

## Discussion

According to the classical views about LTP, long-lasting LTP (1) necessitates the application of several trains of HFS for its induction and (2) is dependent on a synthesis of some proteins, triggered by the inductive stimulus. In the present work, carried out in mouse hippocampal slices, we demonstrated that a long-lasting LTP could be triggered (1) by a single train of HFS and (2) in the presence of inhibitors of protein synthesis.

In order to validate our result, two different inhibitors of protein synthesis (the efficacy of which had been ascertained) were used with two different timings of inhibition and two different periods of recovery after dissection. Except for the independence from protein synthesis, the LTP described in this work shared the classical properties of LTP: it depends on NMDA receptors, α-CaMKII autophosphorylation and PI3-kinase activation. Our results also suggest that high-frequency stimulation induces the insertion of a mixture of GluR2-lacking and GluR2-containing AMPA receptors.

### Long-lasting LTP can be induced by a single train of high-frequency stimulation

In this paper we demonstrated that a single train of HFS could induce a very long lasting LTP. This result was previously obtained in rat hippocampal slices [11], [10], [25] whereas in mice the group of Nguyen has repeatedly shown that a single train (100 Hz, 1 s) induced a short-term LTP lasting only between 1 and 2 h [26]–[29]. First of all, we would like to emphasize the fact that technical conditions have a great influence on the duration of the LTP induced by a single train of stimulation. In previous publications, our group showed that a short-lasting LTP can be transformed into a long-lasting one by modifying the recovery conditions of the slice [30]. We have also observed over years a lengthening of the sustained phase of the LTP induced in mouse hippocampal slices maintained in interface. Indeed, recordings made in 2007 showed a short-lasting LTP going back to baseline within 3 hours [31]. Modifications in the dissection procedure reducing tissue stretching have made it possible to obtain an LTP sustained for 6 hours [32]. And finally improvement in the interface oxygenation and the temperature control, combined with electrode standardization led to an LTP stable for more than 8 hours.

A high level of NMDA activation is probably responsible for the facilitation of the induction of the LTP observed in the present study. Such a high level of NMDA receptor activation could be explained in at least three ways.

First, it could be that previous NMDA receptor stimulation during dissection or recovery would induce a “priming” of NMDA receptors by activity-dependent changes of NR2A/NR2B ratio [33]. Once “primed”, NMDA receptors would allow a larger calcium entry during the single train of stimulation. Our results have ruled out this hypothesis: blocking NMDA activity during dissection and recovery (with 0 Ca^++^ or 2.6 Mg^++^ or by APV) did not modify LTP.

Secondly, NMDA receptor sensitization could be due to an enhanced release of D-serine by astrocytes. NMDA receptor activation needs glutamate to be released by presynaptic terminals in the presence of a co-agonist, glycine or D-serine. It has been demonstrated that in slices, co-agonist sites are not saturated by glycine and that D-serine released by astrocytes is the main endogenous co-agonist of synaptic NMDA receptors [34], [35]. D-serine increases basal NMDA transmission [36] as well as LTP induction [37], [38]. This hypothesis was not proved but astrogliosis and glial STAT3 activation has been demonstrated after slicing in acute slice by Damiani and O'Callaghan [39] while in organotypic slices from mature rats, glial reaction can be observed up to one week after dissection [40].

Thirdly, an increased extracellular glutamate concentration could be responsible for the activation of extrasynaptic NMDA receptors containing NR2B subunit [41]. This activation has been shown to induce postsynaptic depolarization and to increase the excitability of pyramidal cells [42]. Extracellular glutamate concentration is regulated by glutamate transporters present on astrocytes. It has been demonstrated that the activity of glutamate transporters is regulated by the JAK-STAT pathway and can be modified in reactive astrocytes [43]. Perea and Araque [42] showed that high frequency stimulation of the Schaffer collaterals induced an increase in Ca^++^ concentration in astrocytes which in turn elicited NMDA receptor-mediated currents in pyramidal neurons. Although we have not proved this hypothesis, a different manipulation of the slices during dissection or recovery could lead to different glial cell reaction and to different results obtained in different laboratories.

In this paper, we also showed that reducing the number of pulses (from 100 to 15) in the train (at 100 Hz) did not shorten the duration of L-LTP but diminished the amplitude of the potentiation. A link between the number of pulses in the train and the amplitude of the LTP has already been demonstrated in rat hippocampal slices in two previous studies. Volianskis and Jensen [23] observed that 2 hours after its induction the amplitude of the LTP induced by 200 pulses at 200 Hz was larger than that triggered by 8 pulses at 200 Hz. Hernandez et al [44] have compared the amplitude of the LTP recorded during one hour and evoked by 40, 100, 200 and 300 pulses delivered in a train at 100 Hz. They have found that, among the 4 groups, the LTP triggered by 300 pulses was larger than that evoked by 40 pulses.

### Long-lasting LTP induced by a single train is independent from protein synthesis

Since the classic reviews of Davis and Squire [2] and Kandel [45] long-term memory is thought to be ultimately mediated by a de novo protein synthesis. The strongest argument in favour of this hypothesis is that long-term memory is prevented by protein-synthesis inhibitors (such as anisomycin or cycloheximide) when the drugs are administered before or just after the presentation of data to be remembered. Although very popular, this conclusion has recently been questioned [3]–[6]. In the rat, Sharma et al [6] showed that intrahippocampal microinfusion of anisomycin in vivo, at dosages equal to those that have previously been shown to block memory consolidation, severely depressed hippocampal electrical activity. This suppression of neuronal activity, by itself and independently of the concomitant inhibition of protein synthesis, can explain the memory deficits observed in studies using intracerebral anisomycin infusion. In the *Drosophila*, memory is believed to result from synaptic changes occurring in the mushroom body (MB). According to popular views, long-term synaptic plasticity in the MB was believed to be dependent on protein synthesis. This hypothesis has recently been questioned by Chen et al [5]. Using a temperature-sensitive ribosome-inactivating toxin to acutely inhibit protein synthesis during long-term memory consolidation, these authors showed that de novo protein synthesis was not required in the neurons of the MB. However, they found that memory storage during olfactory associative conditioning in *Drosophila* necessitated a de novo protein synthesis in a structure other than the MB, the dorsal-anterior-lateral neurons.

The ability of protein-synthesis inhibitors (and of anisomycin in particular) to prevent the development of the late phase of L-LTP when they were applied around induction, has been reported repeatedly [46]–[48]. This has led to the commonly held hypothesis that stabilizing synaptic plasticity for longer than about 2–3 h required a transitory synthesis of new proteins triggered by the LTP-inductive stimulus [45]. According to the current theory about LTP, repeated high frequency stimulations (HFS), dopaminergic receptor activation [49] or β-adrenergic receptor activation [26] enable the induction of plasticity-related proteins (PRPs) synthesis in the soma or in the dendritic tree. These plasticity-related proteins would allow the persistence of the increase in the strength of the synapses, but only in those made able to capture them. This ability, called “the tag” would be a modification within the dendritic spines as a result of their activation. Tagging of a synapse only requires a weak stimulation (e.g. 21 pulses at 100 Hz, [50]). Once tagged, synapses can “capture” newly synthesized PRPs. This theory is very attractive since it can explain the specificity of synaptic plasticity and heterosynaptic facilitation. Determining the nature of the tag has been the subject of intense study since the first demonstration of its existence [16]. Several studies have proposed diverse protein kinase activities (PKA, MAPK, CaMKII) as suitable candidate mechanisms for the tag [15], [29], [50]. Regulation of local protein synthesis [51], modulation of actin dynamics [52] or input-specific PRP trapping into dendritic spines [53] have also been considered as possible contributors for synaptic capture of L-LTP. Recently, in their revised synaptic tagging and capture hypothesis, Redondo and Morris [54] suggested that trafficking of AMPA receptors from perisynaptic pools to postsynaptic density (PSD) pre-existing slots in response to synaptic activity is the initial step common to short-lasting LTP and long-lasting LTP. Then these newly inserted receptors and their anchoring proteins serve as a tag for the capture of PRP. The recent theory developed by Sacktor [55] emphasizes the role of proteins involved in the trafficking of AMPA receptors to (PKMzeta and NSF) or away from (PICK1 and BRAg2) postsynaptic sites. In the basal state, PICK1 maintains AMPA receptors in a pool located outside the postsynaptic sites and formed by constitutive endocytosis. After LTP induction, newly synthesized PKMzeta links to PICK1. This allows the interaction of AMPA receptors with NSF and, as a result, their transport to the synaptic sites. During the maintenance phase, PKMzeta continues to inhibit endocytosis by preventing PICK1-mediated postsynaptic removal of GluR2. Complementarily, Elhers et al [56] have shown that synaptic activity leads to GluR1 immobilization in the PSD. In this common view of LTP maintenance mechanism, an increased synthesis of PKMzeta could explain a shift of the equilibrium between exocytosis and endocytosis of AMPA receptors leading to the stabilization of AMPA receptors in the postsynaptic membrane.

Our results fit with the hypothesis that LTP results from an insertion of extra AMPA receptors into the PSD. By contrast, they are not in agreement with the hypothesis of a need for a de novo protein synthesis to stabilize the newly inserted receptors. Using α-CamKII mutant mice, we have confirmed that NMDA receptor activation is linked to α-CaMKII autophosphorylation and that this step is required for LTP induction. Activation of NMDA receptors leads to calcium entry, α-CaMKII autophosphorylation and insertion of new AMPA receptors in synaptic sites. The stable binding of autophosphorylated α-CaMKII to the NMDA receptor organizes a structural process that leads to the incorporation of AMPA-receptor-binding proteins into the PSD [57], and to the subsequent anchoring of additional AMPA receptors [58].

As α-CaMKII interacts with GluR1 subunit and increases GluR1-containing AMPA receptor incorporation into synaptic membrane [59], we used specific blockers of GluR2-lacking AMPA receptors to check whether these receptors were upregulated during LTP. It seems not to be the case as the proportion of GluR2-lacking receptors is not affected by LTP. But, as endogenous AMPA receptors in mature hippocampal synapses consist mostly of GluR1/GluR2 heteromers, it is not impossible that the increased response was due to the insertion of a mixture of GluR2-lacking and GluR2-containing AMPA receptors. This hypothesis is in accordance with a role of PI3-Kinase in LTP as we demonstrated that LTP was impaired by the application of wortmannin, a PI3-K inhibitor. PI3-kinase can be activated by calcium/calmodulin after NMDA receptor activation and is involved in GluR2 insertion in postsynaptic membrane through the formation of the GluR2/GRIP1 complex [22].

In mice, Nguyen et al have repeatedly demonstrated the dependence on protein synthesis of the L-LTP after a two-hour recovery period in interface. In rats, Redondo et al [15] and Sajikumar et al [11] demonstrated that it was necessary to wait 4 h after the dissection to be able to observe the protein-synthesis dependence of LTP. In our study, we showed with two different inhibitors and with two different recovery periods that long-lasting LTP could be insensitive to protein synthesis inhibition. We checked that the inhibition of protein synthesis by anisomycin and cycloheximide – at the used dosages – was severe (96% of inhibition). We did not use emetine because at the recommended concentration in slices it only reduced protein synthesis by 50%. We added inhibitors throughout the experiment because we had previously shown that it could influence the LTP maintenance [60], [25]. However, to avoid side effects we also carried out experiments where the drug was stopped after the train. For this experiment, we chose anisomycin because it is less reversible that CHX. In every experiment, the fEPSP-slope potentiation was maintained during at least 8 hours after induction at more than 150%. A similar result was obtained by Abbas et al [61] on young animals (12–20-day-old rats) using a submersion recording chamber. These authors suggested that one of these two factors might be responsible for the difference between their results and the more popular ones. This does not seem to be the case, as we made a similar observation on mature mice (6–10 weeks) using an interface recording chamber.

If there is no need for a synthesis of new proteins at the time of induction, it could be that the PRPs were produced at the moment of dissection or during the recovery period, or even before dissection. By waiting four hours after the dissection in the presence of a protein-synthesis inhibitor, we showed that this was not the case. However, we cannot exclude that proteins synthesized at the moment of dissection, for example by the activation of beta-adrenergic receptors [27], would still be present 4 hours later. In “capture” experiments performed by Frey and Morris [62], there is a temporal window of one or two hours during which PRPs are present and can be captured. In our case, if proteasome activity is reduced, PRPs could stay available for a longer time. At 28°C, protein turn-over could be reduced and only be restored during high frequency test-pulse stimulation. PRPs could also be present in the hippocampus before dissection. It has already been shown that enrichment could improve memory and LTP in mice. Following guidelines for experimental animal use, mice are always kept in groups of 5 to 7 in the presence of pipes to play and hide.

The molecular identity of the PRPs is unknown. ARC, Homer1a, PKMzeta and BDNF have been proposed as specific candidates. However, it is possible that the newly synthesized proteins only serve to replenish the pool of worn-out proteins. ARC and Homer 1A have repeatedly been highlighted in the literature as proteins synthesized in response to neuronal activity (in epilepsy or in response to L-LTP-inducing stimuli) [63]–[65]. Homer 1A is involved in AMPA receptor endocytosis and Arc regulates actin polymerization which can modulate receptor trafficking to and from the synapses. However, the results of French et al [66] have cast a doubt on their role in LTP. These authors found that Arc and Homer 1A were transcribed in the dentate gyrus after seizures, after induction of LTP in vivo but not following LTP induction in hippocampal slices. Worse, in the CA1 region, transcription of Arc and Homer is increased after electroshock-evoked seizures but not after LTP induction in vivo or in slices [66]. PKMzeta protein has also been repeatedly identified as a PRP necessary for the maintenance of LTP. The synthesis of this protein increases after the induction of LTP and its inhibition (by ZIP) destabilizes LTP [67], [68]. But, recently the inhibitory effect of ZIP in slices has been questioned [69] and Mei et al [70] have demonstrated that co-application of BDNF and of theta-burst stimulation (TBS) on hippocampal slices triggered both an L-LTP and an increase in the PKMzeta level even in the absence of protein synthesis. These results suggest that BDNF sustains L-LTP through PKMzeta in a protein synthesis-independent manner, revealing an unexpected link between BDNF and PKMzeta.

Finally, there are also reasons to question the candidature of BDNF as a PRP whose synthesis would be triggered by the L-LTP inductive stimulus. Indeed, Pang et al. [71] demonstrated that the late phase of L-LTP could become insensitive to anisomycin when BDNF (brain-derived neurotrophic factor) was applied to the slices. In other studies BDNF has been shown to be necessary and sufficient to induce LTP [72]. However, BDNF can be released by glial cells (astrocytes and oligodendrocytes, [73]) and ischemia can modify BDNF expression by activating glial cells [74]. BDNF production can also be regulated by the level of tPA in the hippocampus. It has been demonstrated that prenatal stress reduces the level of the mature form of BDNF (mBDNF) in the hippocampus of young rats (5 weeks) through a reduction of tPA release [75].

### Conclusion

At the end of a recent review about the question of the protein synthesis-dependence of L-LTP maintenance, Abraham and Williams [76] concluded that the case was still very strong for the necessary role of protein synthesis in LTP, believing though that the issue had not been fully explored. Here, despite using a “strong” protocol for applying the protein-synthesis inhibitors (high dosage, application of the drug around induction and till the end of the experiment), we were able to elicit an L-LTP that was insensitive to protein-synthesis inhibitors.

## Materials and Methods

### Slice preparation

All animal procedures were carried out in accordance with National Institutes of Health regulations for the care and use of animals in research and with the agreement of the local ethics committee. The board of the ethics committee of the University of Mons specifically approved this study (Project number GO/01/03). The experiments were performed on transverse hippocampal slices (400-µm thickness) from C57BL/6 mice (6–10 weeks-old) and for some experiments from *αCaMKII-T286A* mice generated as described [77]. The animals were anaesthetized with halothane and then decapitated. The hippocampus was isolated and sliced using a McIlwain chopper. The slices were incubated in an interface chamber (FST, Vancouver, Canada) at 28°C. For long lasting (8 hours) experiments, we used the Edinburgh temperature control system [15], [78]. This system maintains a constant and uniform temperature inside the whole experimental rig, a procedure that prevents condensation droplets from falling onto the slices in the interface chamber. If not otherwise specified, the slices were perfused (1 mL/min) with artificial cerebro-spinal fluid (ACSF) containing 124 mM NaCl, 4.4 mM KCl, 26 mM NaHCO_3_, 1 mM NaH_2_PO_4_, 2.5 mM CaCl_2_, 1.3 mM MgSO_4_ and 10 mM glucose, aerated with 95% O_2_, 5% CO_2_. Carbogen consumption was 250 mL/min.

### Electrophysiological Recordings

Field excitatory post-synaptic potentials (fEPSPs) were recorded with a glass microelectrode (2–5 Mohm, filled with ACSF) positioned in the stratum radiatum of area CA1. Two bipolar cluster electrodes (FHC, Bowdoin, USA) were used to stimulate two distinct bundles of Schaffer collaterals (Stimulations 1 and 2, S1 and S2, Fig. 1A1, A2). The slices rested for at least 1 h30 before positioning of the electrodes and determination of the stimulation parameters. Baseline recording was then performed for 30 minutes. Field EPSPs were recorded at 40% of the maximum amplitude obtained in an input-output curve. Test stimulation was a biphasic pulse (0.08 ms pulse duration per half-wave) delivered 1/60s.

### Induction of Long-Term Potentiation

LTP was triggered in one of the two channels (S1) at least 2 h30 after dissection by applying a single 100 Hz train at test strength (1 sec, 0.15 sec or 0.25 sec) or by four 100 Hz trains spaced by 5 minutes. The other channel (S2) served as a control.

### Data Analysis

Stimulation, data acquisition and analysis were performed using the WinLTP program [79] (Website: www.winltp.com). For each slice, the fEPSP slopes were normalized against the average slope over the 30 minutes preceding LTP induction. For all the experiments referred to in the present work, the late phase of L-LTP was assessed by analyzing the mean slopes of the fEPSP measured at different times after LTP induction. Student t-test and One Way Anova for more than two samples were used. In the results section data were presented by the mean ± SEM.

### Drug Treatment

Cycloheximide (Sigma) (300 μM), 2-amino-5-phosphonovaleric acid (APV, Tocris) (50 µM), emetine (Calbiochem) (20 µM) and 1-naphthyl acetyl spermine trihydrochloride (NASPM, Tocris) (50 µM) were dissolved in ACSF. Anisomycin (Sigma, 25 and 40 µM), forskolin (Alomone, 50 µM), IBMX (Sigma, 30 µM) and wortmannin (Tocris, 20 µM) were dissolved in DMSO and diluted down to reach their final concentration (in 0.1% DMSO).

### Measurement of [^3^H] Leucine Incorporation into Proteins in Slices

The level of protein synthesis inhibition in the slices was measured by incorporating [^3^H] leucine into trichloroacetic acid (TCA)-precipitable macromolecules [80]. For incorporation, L-[4,5-^3^H]leucine containing ACSF was added for 1 h. At the end of this period, L-[^1^H]-leucine containing ACSF was added for 20 min in order to remove non-incorporated L-[4,5-^3^H]leucine. Then the slices were removed from the interface chamber. L-[4,5-^3^H] leucine (GE Healthcare, Belgium) with a specific activity of 160Ci/mM was used at a concentration of 1 µCi/ml. It was provided in solution and directly diluted in ACSF. L-leucine (Sigma) was first dissolved in 2% ethanol solution and then diluted at 1 µg/ml.

Seven slices from the same hippocampus were treated simultaneously. Once they were made, the slices were immediately treated with 80 µL extraction buffer (0.1 M hepes, 0.1% triton) containing a cocktail of protease inhibitors (Roche Diagnostics, USA) and then frozen at −80°C until use.

Mechanical extraction of the proteins was performed in the extraction buffer at 4°C. After centrifugation (800G, 10 min, 4°C), proteins of the supernatant were precipitated with ice-cold TCA 20%. After a second centrifugation (800G, 10 min, 4°C), the pellet was washed twice with ice-cold TCA 10%. Measurement of the percentage of incorporation of L-[4,5-^3^H] leucine in the pellet dissolved in KOH 1M was carried out using a scintillation counting system.

A small part of each sample was used for protein quantification using the Non-Interfering Protein Assay (Calbiochem, USA) protocol. The radioactivity of each sample was balanced with total protein concentration. The percentage of inhibition of [^3^H] leucine incorporation produced by drug treatments was calculated by comparing incorporation in treated slices with that in control slices, the measurements being performed in both groups at the same time after dissection.
